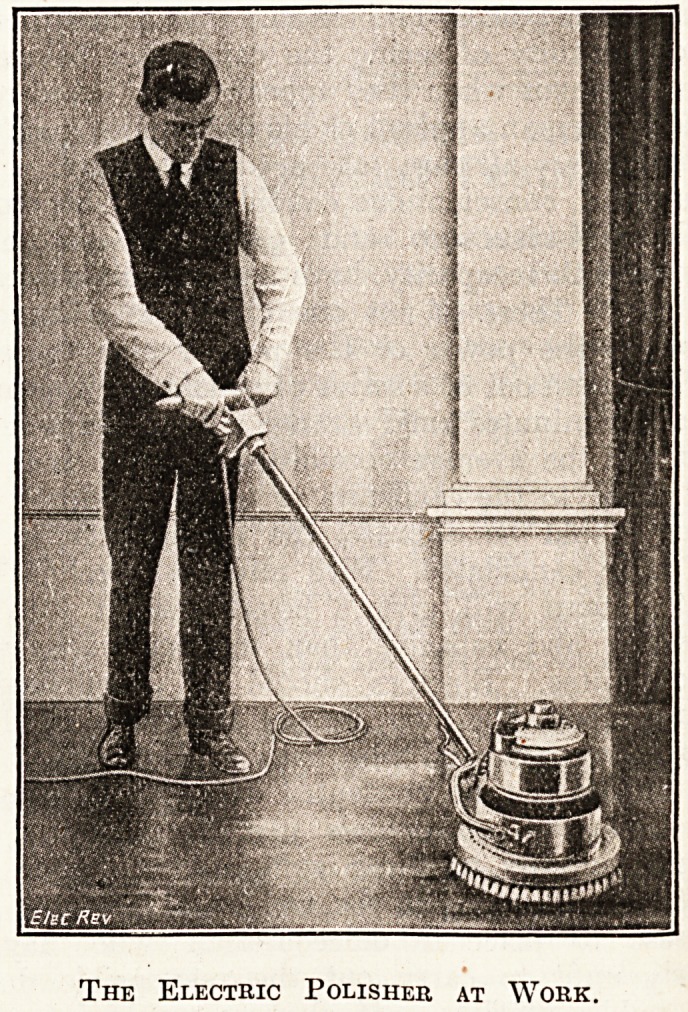# Institutional Needs

**Published:** 1914-12-05

**Authors:** 


### INSTITUTIONAL NEEDS.

### THE ELECTRIC FLOOR POLISHER.

Two problems present themselves in the case of hospital floors. The first is how best to polish them; and the second how they can, when worn, be renovated. The Electric Floor Polishing Machine Company, 118 City Road, E.C., claims to answer satisfactorily these two questions, by stating that they can make old and even badly worn floors equal to new, and can give a much finer and smoother finish by their electric polisher than can possibly be given by hand-work. The accompanying block shows the latter in use. Its rotary action secures a smooth and level finish, and an even surface is produced, while it is intended both for wood and composition floors. Being made adaptable by various attachments suited for different purposes, such as brushes, sand-paper holders, grinding and surfacing plates (for floors badly worn and splintered), the machine contains in itself a minor workshop, and possessing a small motor is operated from the

The Electric Polisher at Work.

existing electric-light installation. For large areas of floors requiring different treatment, some to be polished and some to be surfaced, this composite equipment has obvious advantages. Now that the firm has a contract department, where all branches of work are undertaken, men and machines can be sent anywhere at short notice. It is understood that one of the Electric Floor Polishing Machine Company's latest orders has been carried out at the Norwich Isolation Hospital, "where the floors were considered to be past repair," and that other institutions, including Bexley Asylum, provide examples of their work. The firm's claims are specially noteworthy in that floors are sometimes changed at the instigation of a particular official, who from his or her position may prefer, say, staining to scrubbing, or *vice versa*, and, if afterwards this preference prove unsatisfactory, land the institution in a degree of expense, which the successful employment of the above machine might well minimise and make renovation swift and relatively easy.

## Figures and Tables

**Figure f1:**